# A systematic review of hypervirulent *Klebsiella pneumoniae* research: bibliometric and topic modeling perspectives

**DOI:** 10.3389/fmed.2025.1545678

**Published:** 2025-04-11

**Authors:** Tian Tian, Hui Han, Zhi-Hui Guan, Ke Zhang, Xinghe Huang, Wenyue Wang, Xuan Zhang, Fei Zhang, Leijia Wei, Xin Zhang, Jia-He Wang

**Affiliations:** ^1^Department of Family Medicine, Shengjing Hospital of China Medical University, Shenyang, China; ^2^Science and Technology Research Center of China Customs, Beijing, China; ^3^Department of Developmental Cell Biology, Key Laboratory of Cell Biology, Ministry of Public Health, China Medical University, Shenyang, China; ^4^College of Metrology and Measurement Engineering, China Jiliang University, Hangzhou, China; ^5^Department of General Practice, The First Hospital of China Medical University, Shenyang, China; ^6^Department of Infection Diseases, The First Affiliated Hospital of China Medical University, Shenyang, China

**Keywords:** hypervirulent *Klebsiella pneumoniae*, *Klebsiella pneumoniae*, hypervirulence, antimicrobial resistance, bibliometrics, topic modeling, epidemiological characteristics

## Abstract

**Background/objective:**

Hypervirulent *Klebsiella pneumoniae* (hvKP) is an emerging global health threat, exhibiting increased virulence and multidrug resistance compared to classic *K. pneumoniae*. Understanding the research landscape surrounding hvKP is crucial for developing effective control strategies. This study aimed to comprehensively analyze the global research trends in hvKP from 2013 to 2024 using bibliometric and topic modeling techniques.

**Methods:**

Data from 1,014 articles on hvKP, retrieved from the Web of Science Core Collection, were analyzed using Bibliometrix, CiteSpace, and VOSviewer to assess publication trends, collaborations, geographical distribution, and keyword co-occurrence. Latent Dirichlet Allocation (LDA) topic modeling was employed to identify key research themes.

**Results:**

The analysis revealed a steadily increasing volume of hvKP research, with China and the United States as major contributors. Four primary research themes emerged: high virulence phenotypes and mechanisms; drug resistance and treatment strategies; genetic and molecular mechanisms; and epidemiological and transmission characteristics. Research hotspots included virulence mechanisms, drug resistance, genomic detection approaches, and epidemiological features.

**Conclusion:**

This bibliometric analysis provides a comprehensive overview of hvKP research, highlighting key trends and research gaps. The identified research hotspots inform future research directions and contribute to the development of effective strategies for combating hvKP infections. The increasing research volume underscores the urgent need for continued investigation into this significant public health threat.

## 1 Introduction

Hypervirulent *Klebsiella pneumoniae* (hvKP) is a hypervirulent (hypermucoviscous) variant of *K. pneumoniae*, distinct from classic *K. pneumoniae* (cKP). It represents an evolving pathotype characterized by enhanced virulence (1). Early reports emerged from Taiwan in the mid-1980s and 1990s, describing a Klebsiella species associated with a unique clinical syndrome, causing liver abscesses as a sole pathogen (2). Subsequently, many reports referred to it as hypermucoviscous *K. pneumoniae* due to the hypermucoviscous colony morphology observed on specific agar plates (3). In 2004, Taiwanese researchers identified a novel gene, magA (“mucoviscosity-associated gene A”), in hypermucoviscous *K. pneumoniae* strains (3), initially considered a hallmark gene for hvKP (4–7), leading to initial ambiguity in hvKP definition and research. Subsequent studies revealed that not all hypermucoviscous strains carry magA (8), and many hvKP strains exhibit non-K1/K2 capsular serotypes (9, 10). In 2013, Alyssa et al. comprehensively defined hvKP based on its characteristic features (11), formally establishing the term hypervirulent *K. pneumoniae* (hvKP). However, the precise definition of hvKP remains a subject of ongoing refinement due to its diverse genetic background and complex virulence mechanisms (12).

HvKP is predominantly prevalent in the Asia-Pacific region, with colonization rates in healthy adults ranging from 2.7% in Thailand to 16.7% in Japan (13). While the Asia-Pacific remains the epicenter of hvKP infections, increasing case reports from Europe and North America highlight its expanding global footprint (14, 15). Large-scale genomic analyses have identified multiple concurrent antibiotic-resistant clusters of hvKP strains worldwide, underscoring its accelerating global spread (16).

Clinically, hvKP is associated with severe infections, including pneumonia, bloodstream infections, and even brain abscesses, leading to high morbidity and mortality (17). For instance, a case report from Japan documented a diabetic patient who developed emphysematous cholecystitis and disseminated infection due to hvKP K2-ST65, ultimately resulting in fatal multi-organ failure (18). Similarly, another study reported a case of community-acquired pneumonia caused by hvKP K2-ST86, where the patient rapidly deteriorated and died (19).

The convergence of hypervirulence and antimicrobial resistance has emerged as a defining evolutionary trajectory of *K. pneumoniae*, posing a significant and emerging public health threat (20). Retrospective studies have shown a marked increase in hvKP infections over recent years, closely linked to patterns of antimicrobial resistance (21). In China, carbapenem-resistant hvKP (CR-hvKP) strains have been widely reported across multiple regions (22), with regional dissemination patterns exemplified by the ST25 CR-hvKP strains isolated in central-southern China (23). Hospital outbreaks of multidrug-resistant hvKP, such as the ST11 strain, further highlight the dual threats of virulence and resistance evolution (24, 25).

Despite three decades of extensive basic and clinical research since its discovery, our understanding of hvKP remains incomplete. Key challenges include the lack of objective diagnostic tests, which hampers accurate prevalence estimation; unclear genotype/phenotype markers, leaving the mechanisms of infection partially understood; and insufficient data to determine optimal antimicrobial therapies for hvKP infections (26, 27). Current research predominantly consists of scattered case reports and small-scale studies, lacking systematic integration and comprehensive analysis.

This study employs bibliometric analysis and topic modeling approaches to comprehensively examine the research domain of hvKP. With advancements in bibliometric software and scientific databases (28), researchers have increasingly utilized relational bibliometrics for network analysis (29), which offers robust analytical capabilities for uncovering macro-level research trends and knowledge structures. Here, we analyzed literature metadata (e.g., authors, journals, and keywords) to construct knowledge maps, revealing the overall structure and key nodes in hvKP research. Data were sourced from the Web of Science Core Collection and analyzed using tools such as Bibliometrix, CiteSpace, and VOSviewer for visualization. However, bibliometric analysis primarily focuses on quantitative relationships and co-occurrence patterns, limiting its ability to explore thematic content and research directions in depth. To address this, we integrated topic modeling methods, employing the Latent Dirichlet Allocation (LDA) model from the Python Gensim library to perform topic mining on literature abstracts. As an unsupervised machine learning technique, the LDA model identifies latent themes within texts and represents each topic using word probability distributions (30, 31). During preprocessing, we applied stopword removal, punctuation removal, and stemming to enhance model accuracy and efficiency. After evaluating models with varying numbers of topics, the optimal theme model was selected.

## 2 Materials and methods

This study selected the Web of Science Core Collection (WoSCC) as the data source, given its comprehensive coverage of high-quality research publications. The search covered all WoSCC editions, including the Science Citation Index Expanded (SCI-EXPANDED), Social Sciences Citation Index (SSCI), Current Chemical Reactions (CCR-EXPANDED), and Index Chemicus (IC). The search query was “((((TS = (hypervirulent *Klebsiella pneumoniae*)) OR TS = (hvKP)) OR TS = (hypermucoviscous *Klebsiella pneumoniae*)) OR TS = (hypervirulent *Klebsiella pneumoniae*)) OR TS = (hypermucoviscous *Klebsiella pneumoniae*))),” with a time span from 2013 to 2024, resulting in 1,147 records.

### 2.1 Inclusion and exclusion criteria

#### 2.1.1 Inclusion criteria

1. Document types include research articles and review articles.
2. Documents are published in English.
3. The content directly relates to research on hypervirulent *Klebsiella pneumoniae* (hvKP).
4. The publication date is between 2013 and 2024.

#### 2.1.2 Exclusion criteria

1. Document types such as book reviews, editorials, news articles or conference papers.
2. Documents published in languages other than English.
3. Documents with irrelevant or low relevance to hvKP.
4. Duplicate publications or records in the database.

**Figure 1:**
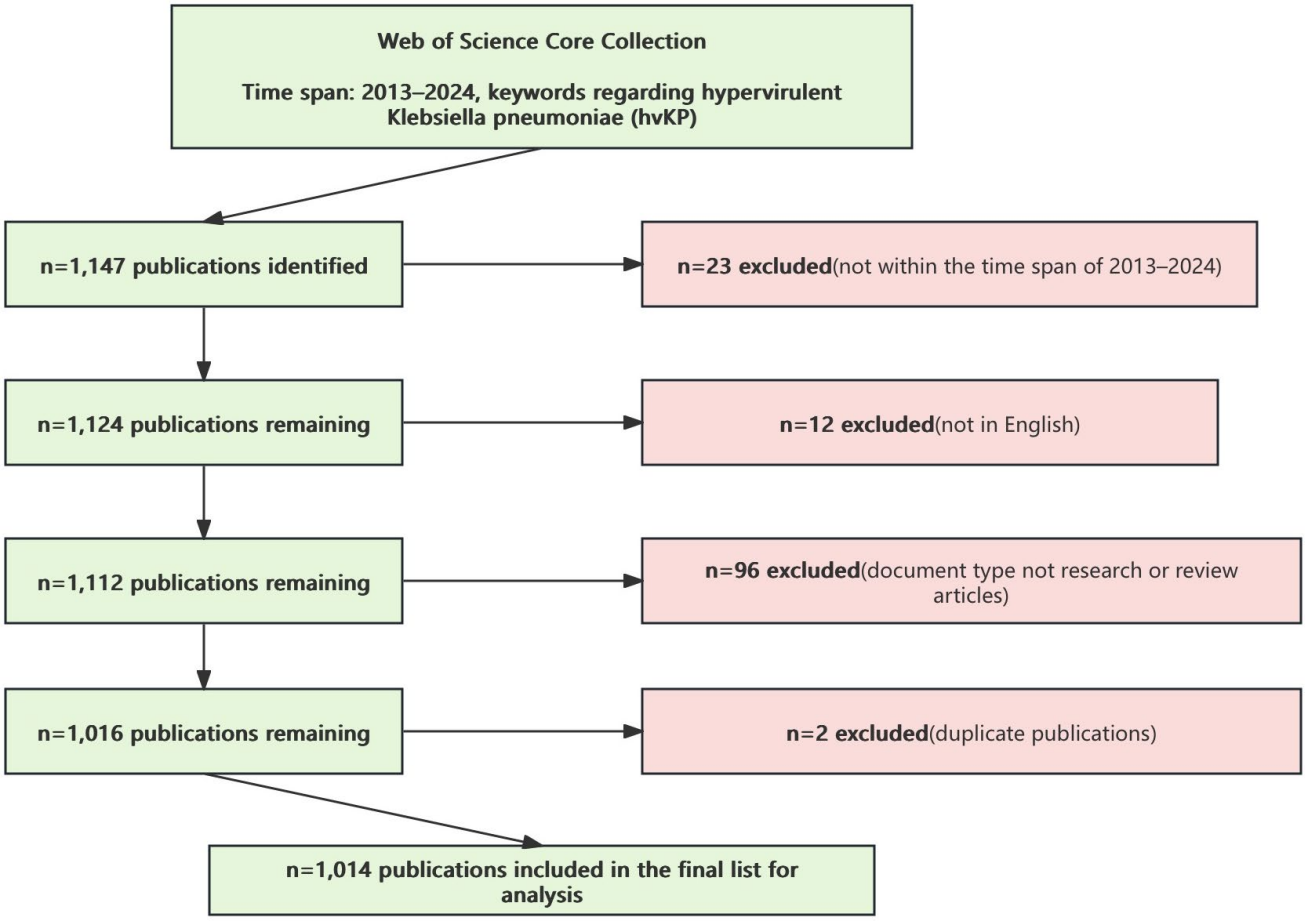
Flowchart of literature inclusion process for hypervirulent *Klebsiella pneumoniae* (hvKP).

After excluding irrelevant document types, languages, and duplicates, a total of 1,014 publications were included (Figure 1).

### 2.2 Data export and processing

The data were exported as plain text files, including full records and cited references. The cleaned data were imported into the following tools for analysis and visualization.

- *Bibliometrix 4.3.1*: Used for analyzing annual publication output, citation counts, authors’ h-index, publication trends over time, three-field plots, and document co-citation analysis.
- *CiteSpace 6.1.R6 (64-bit) Advanced*: Used for generating structural co-occurrence maps, journal dual-map overlays, and keyword analysis.
- *VOSviewer 1.6.19*: Used for author co-authorship analysis and country network visualization.
- *LDA topic modeling*: For LDA topic modeling, the LdaModel class in the Gensim library was employed, supporting online, constant memory, and distributed training for efficient large-scale corpus processing. The core estimation code is based on the onlineldavb.py script by Matthew D. Hoffman, David M. Blei, and Francis Bach: “Online Learning for Latent Di-richlet Allocation,” NIPS 2010. Preprocessing involved text cleaning, including synonym merging and phrase recognition, and Porter Stemming for stemming. The model was trained using the preprocessed document vectors. The optimal number of topics (k) was determined to be 4 by evaluating model coherence and perplexity across a range of 2–15 topics. Model parameters were set as follows: num_topics = 4; other parameters used default values (alpha = ‘symmetric’, eta = None, decay = 0.5, offset = 1.0, etc.). The model was trained incrementally using the LdaModel’s update() method, saved and loaded using save() and load() methods, respectively, and new document topic distributions were inferred using the getitem method.

## 3 Results

### 3.1 Publication and citation trends

**Figure 2:**
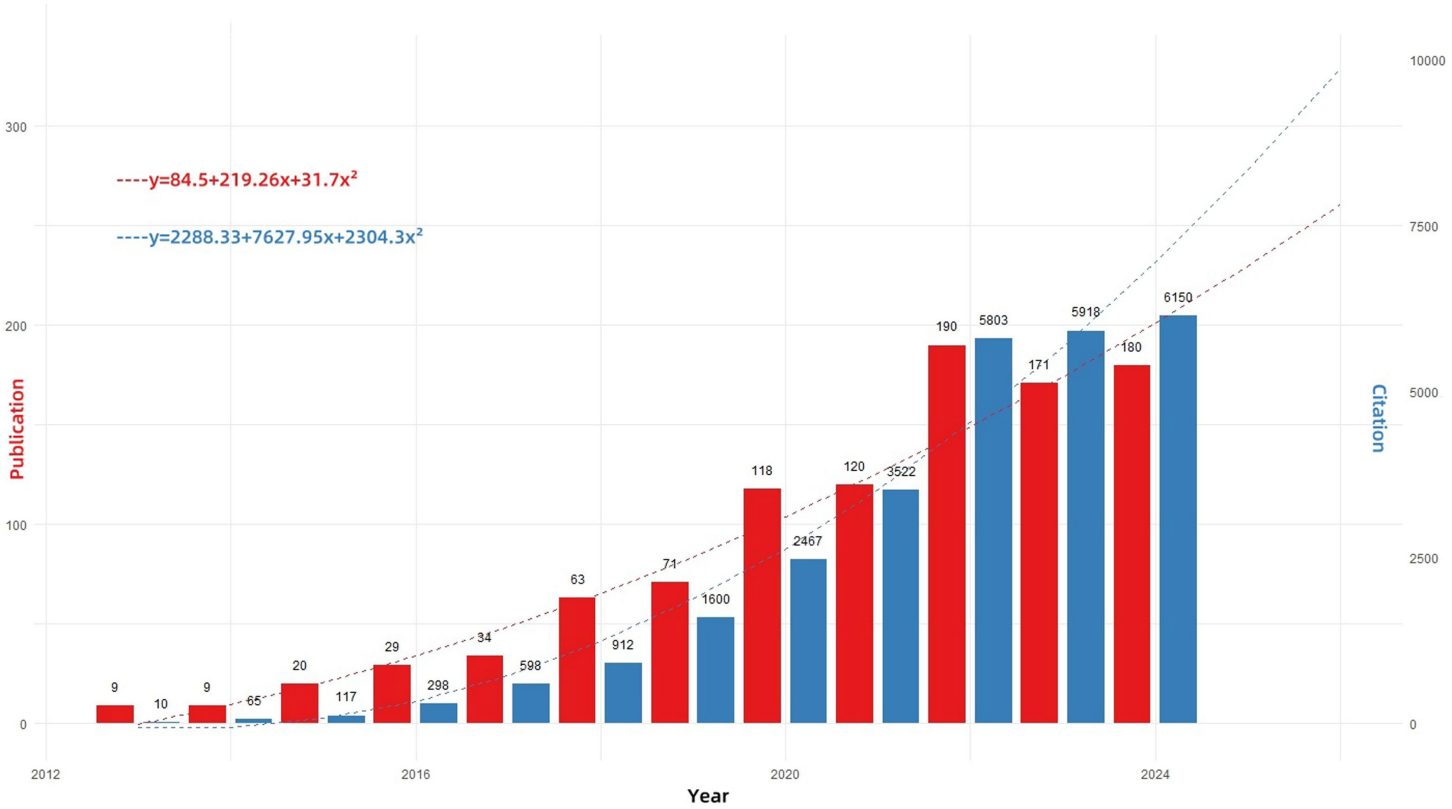
Annual publications and MeanTCperYear in the research field from 2012 to 2024. “Publications (red)” indicates the annual number of papers published; “MeanTCperYear (blue)” (mean total citations per paper divided by the number of citable years) is a useful metric to assess the yearly impact of this research field.

Figure 2 illustrates the trends in the number of publications and citations related to hypervirulent *Klebsiella pneumoniae* (hvKP) from 2013 to 2024 (data up to November 2024). The results demonstrate a consistent yearly increase in both publication output and citation counts for hvKP-related research. To project future trends, we fitted quadratic polynomial regression models to the publication and citation data, yielding the following predictive equations: Publication count: y = 84.5 + 219.26x + 31.7x^2^; Citation count: y = 2288.33 + 7627.95x + 2304.3x^2^ (where x represents the year, with 2013 as year 0). These equations can be used to estimate future publication and citation counts for hvKP research. Before 2013, the annual publication count was consistently ≤7. The sharp increase from 2013 onwards is likely attributable to the expanding geographical distribution of hvKP. During this period, hvKP emerged as an important pathogen in various regions, including the United States, Canada, Europe, Israel, South Africa, and Australia (32), with infections no longer confined to individuals of Asian descent or those with recent travel history to Asia (33). This broadened recognition of hvKP as a globally significant cause of lethal pneumonia fueled increased research interest. The quadratic polynomial regression models predict the continued robust growth of hvKP research. Sustained attention and investment in hvKP research are crucial for safeguarding global public health.

### 3.2 Author analysis

**Figure 3:**
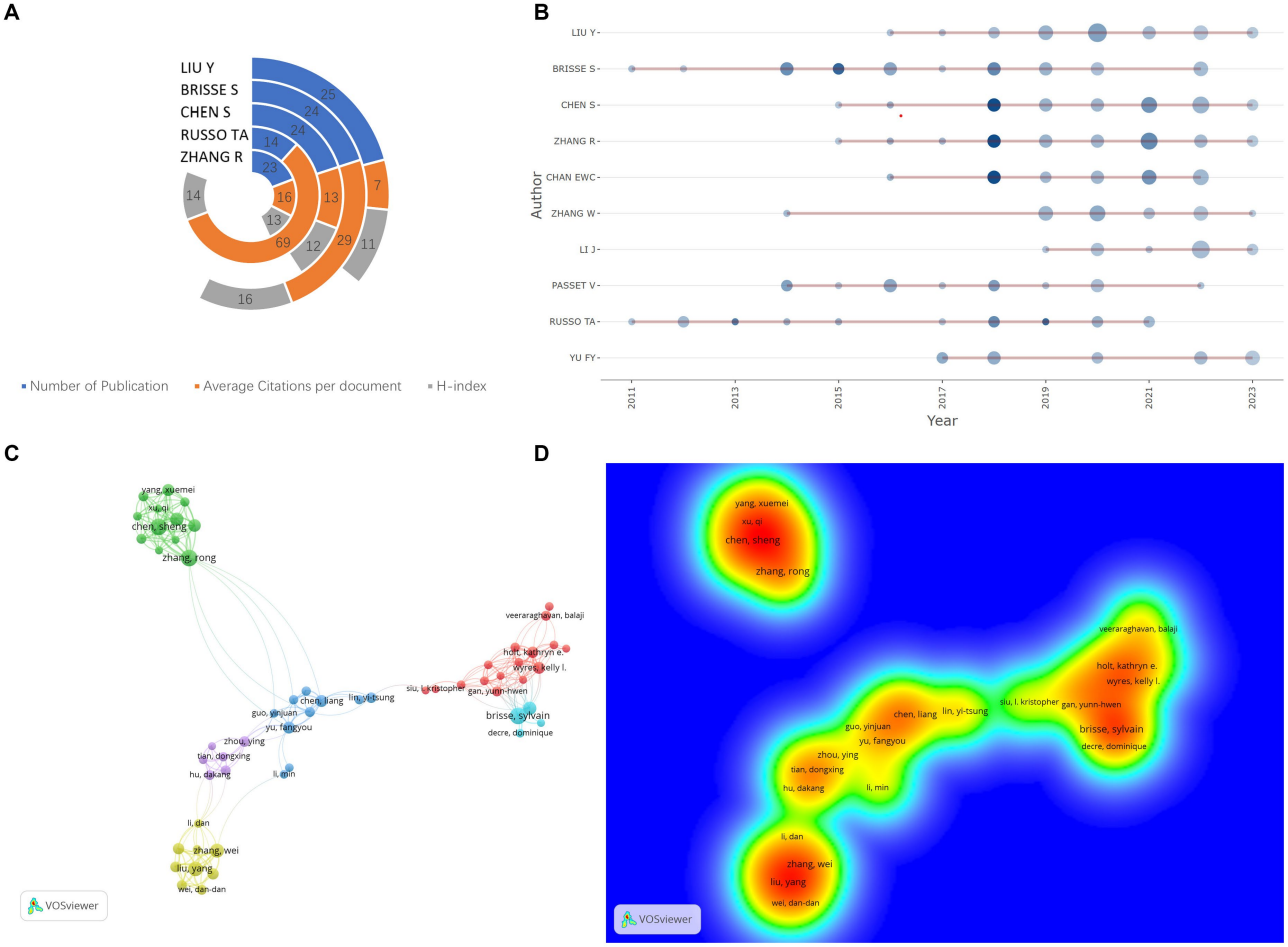
**(A)** Academic impact metrics of top 5 authors: Total publications, average citations per document, and H-index [Quantifying scholar influence by balancing publication count and citation impact (114)]. **(B)** Authors’ temporal productivity [Dot size reflects annual article count, shading intensity denotes Total Citations per Year (TCpY)]. **(C)** Co-authorship network analysis; **(D)** Co-authorship density visualization.

Figure 3A presents key metrics for the top five most prolific authors in the hvKP research field, including publication count, average citation count, and h-index (34). Liu, Yang and Sylvain Brisse stand out as the most prolific and highly cited authors, respectively, demonstrating significant influence in the field. Liu, Yang from Nanchang University focuses on rapid molecular detection of hvKP (35), antimicrobial resistance mechanisms [e.g., carbapenem resistance (36) and ceftazidime/avibactam resistance (37)], and infection pathogenesis (38). Sylvain Brisse from the Institut Pasteur, Paris, was recognized as a “Highly Cited Researcher in the field of Microbiology” in 2020, and his research primarily centers on hvKP genomics (39, 40). Figure 3B displays the publication timelines for the top 10 most prolific authors. Zhang, Rong (Zhejiang University), Edward Wai-Chi (City University of Hong Kong), and Chen, Sheng exhibited a peak in publications in 2018, indicating a significant surge in their influence and recognition within the hvKP research community that year. This may be linked to their collaborative work on three publications in 2018 focusing on carbapenem-resistant hypervirulent *K. pneumoniae* ST11 (25, 41), facilitated by an outbreak at an affiliated hospital of Zhejiang University in China, allowing for detailed epidemiological and microevolutionary analyses. These three authors also form the most densely connected research group (warmest color tones in Figure 3D), with Chen, Sheng playing a crucial role in information dissemination and collaboration (the center of the green collaboration module in Figure 3C). Their collaborative work centers on the epidemiology and transmission patterns of hvKP (42) and the study of hvKP virulence plasmids (43, 44).

### 3.3 Institutional analysis

**Figure 4:**
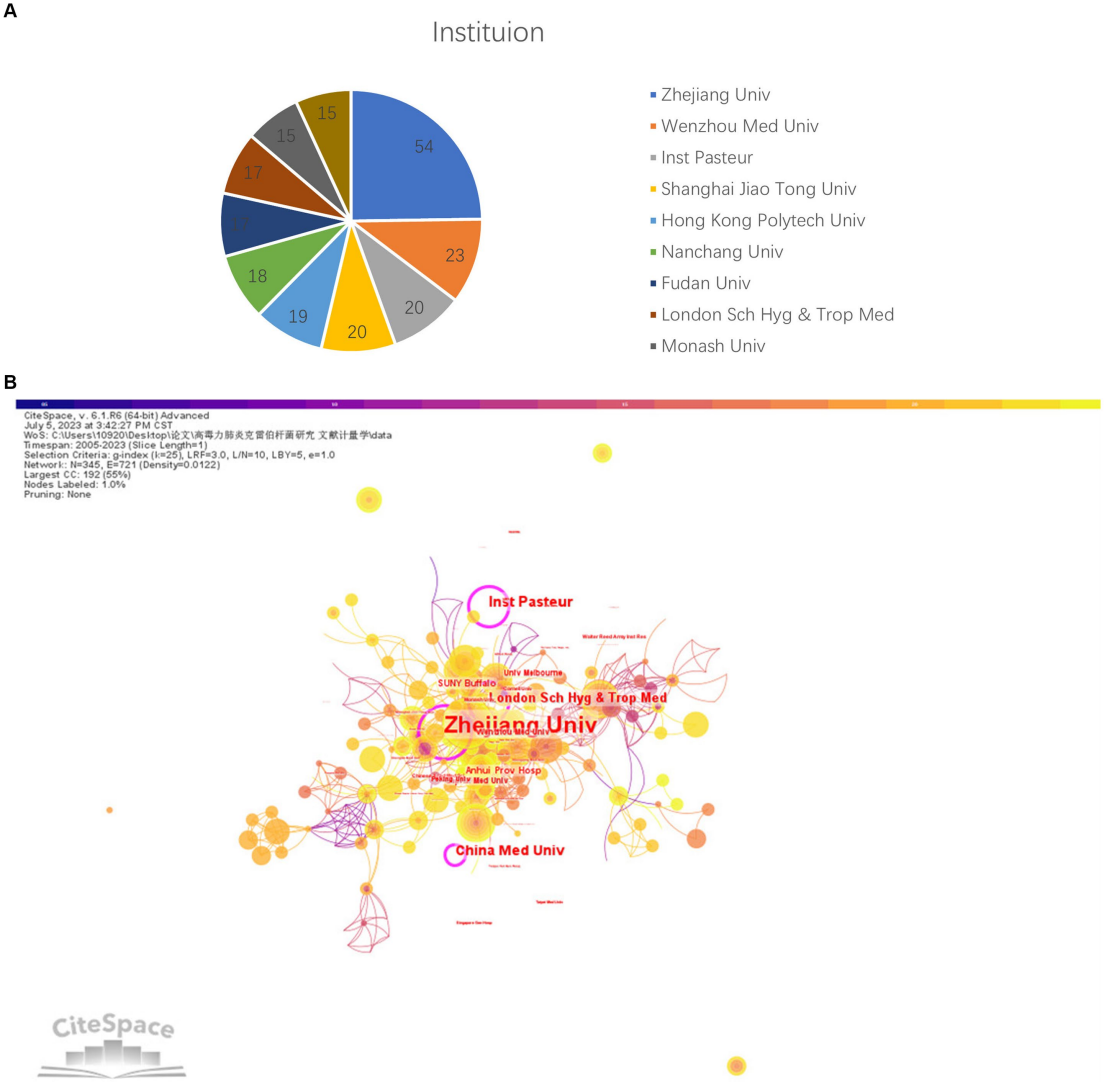
**(A)** Top 10 institutions with the highest number of papers. **(B)** Inter-institutional Collaboration Network [Purple node periphery denotes high betweenness centrality (BC ≥ 0.1), reflecting bridging frequency in shortest-path connections (115)].

Zhejiang University is the leading contributor, with 54 publications. Other contributing institutions include Wenzhou Medical University (23), Shanghai Jiao Tong University (20) (Figure 4A). Figure 4B depicts the institutional collaboration network for hvKP research. A total of 345 institutions participated (*N* = 345), with 721 collaborations (*E* = 721) and a network density of 0.0122, close to zero, indicating a relatively low overall network density. However, several tightly knit sub-networks exist, notably those centered around Zhejiang University (Count = 54, BC = 0.20) and the Institut Pasteur (Count = 20, BC = 0.12). These institutions not only exhibit high publication output but also high BC, signifying the formation of strong collaborative groups within the hvKP research field.

### 3.4 Country analysis

**Figure 5:**
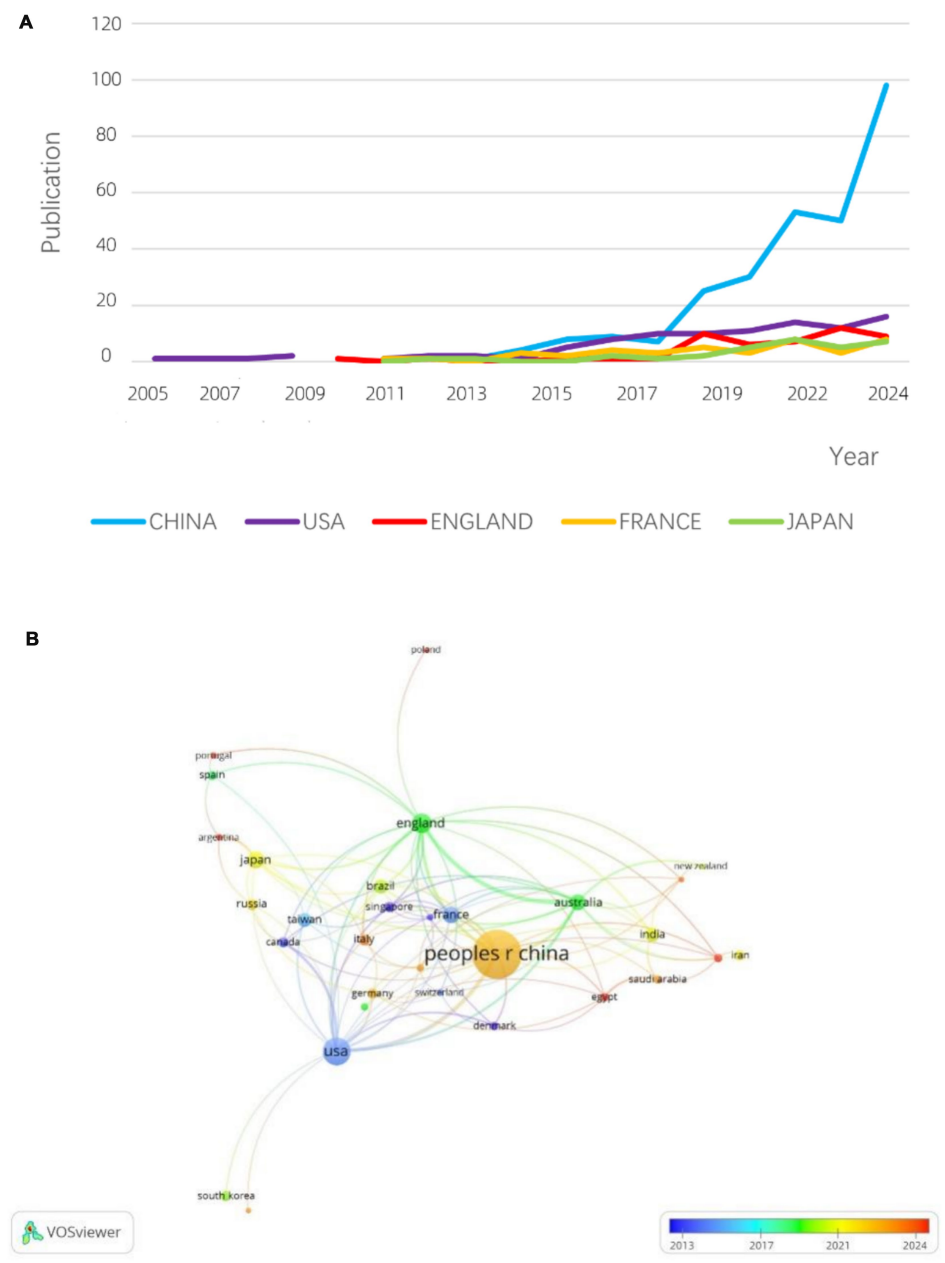
**(A)** Top 5 countries’ production over time. **(B)** Country network visualization.

Figure 5A presents the publication timelines for the top five countries/regions. Before 2011, the United States and the United Kingdom dominated hvKP research. However, after 2017, China experienced exponential growth in hvKP-related publications, surpassing other countries/regions. This may be attributed to China’s large population and its longstanding status as a high-prevalence region for hvKP infections. A 2016 study in China reported that 30.9% of *K. pneumoniae* isolates tested positive using the string test (45). Figure 5B shows the inter-country/region collaboration network. The United States and China exhibit the most frequent collaborations, although the collaborative output is relatively low compared to their individual outputs, suggesting significant potential for further collaboration. The UK and Australia show a strong collaborative relationship, with 16 joint publications representing 47% of Australia’s total output, providing a model for other countries.

### 3.5 Journal analysis

**Figure 6:**
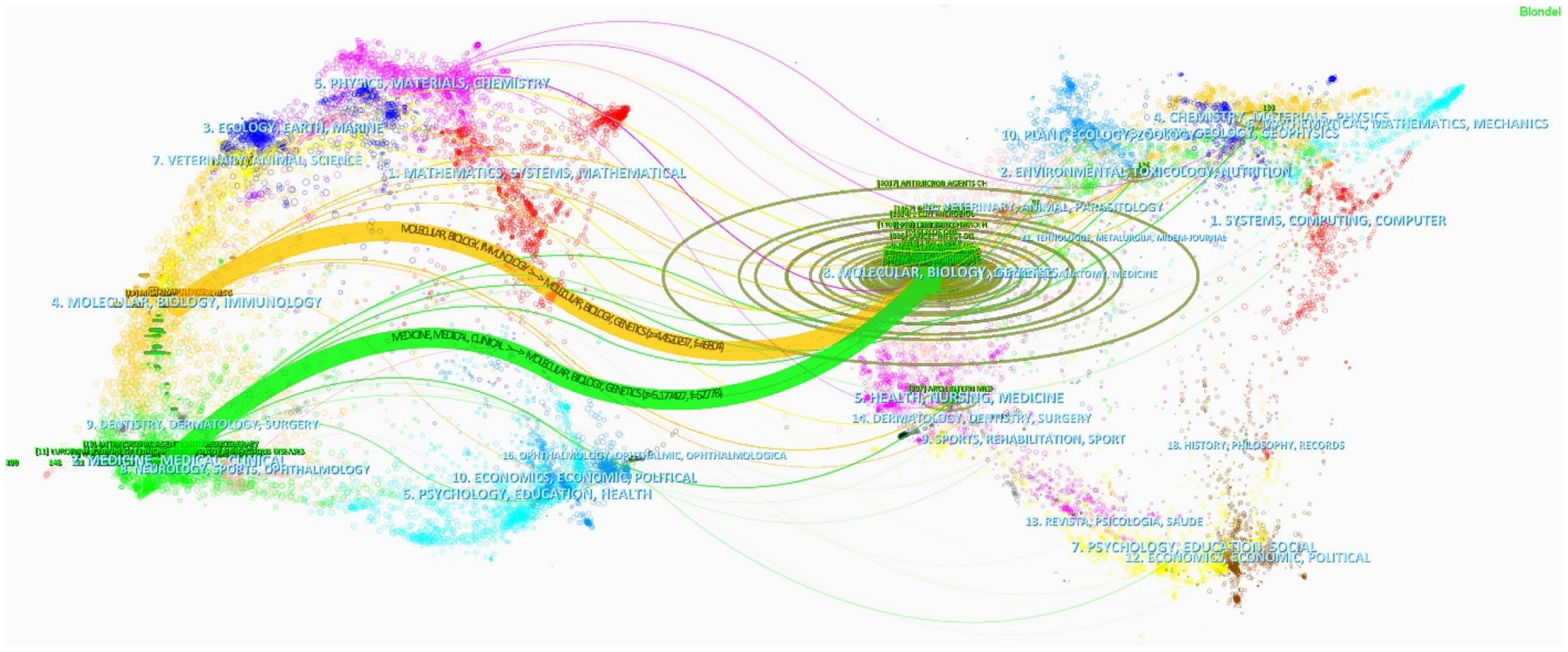
Dual-map overlay of journals.

Supplementary Table S1 reveals that the driving force behind hvKP research is distributed across numerous Q2 journals. While Frontiers in Cellular and Infection Microbiology is the only Q1 journal among the top 10, its substantial publication count (third place) indicates significant research output. This suggests that the field encompasses both high-impact research and a broad base of in-depth investigations, providing a solid foundation for future advancements. Figure 6 displays a dual-map overlay of journals, showing that journals in immunology and clinical medicine are predominantly cited by journals focused on molecular biology, genetics, and microbiology. This highlights the importance of interdisciplinary integration in advancing research, exemplified by studies exploring the impact of genetic variations on hvKP-host interactions and pathogenesis (46), and the role of hvKP’s molecular mechanisms of high mortality in informing effective clinical treatments (47).

### 3.6 Highly cited publications

This study identified the top 10 most frequently cited articles to analyze the current research trends in hvKP (Supplementary Table S2). The two most frequently cited articles, both authored by Russo, Thomas A., are review papers on hvKP. Russo’s 2013 review (11) was the first to define this emerging strain as hypervirulent *K. pneumoniae* (hvKP), focusing on its pathogenic mechanisms, epidemiological characteristics, and diagnostic and therapeutic approaches. His 2019 review (1) further explored the pathogenesis, colonization, and infection processes of hvKP, emphasizing the role of virulence factors. The third most cited article, a 2016 review by Paczosa and Mecsas (48), primarily discussed the K-antigen of hvKP. A 2012 review by Siu et al. (49) highlighted the unique invasive syndrome caused by hvKP, particularly its association with liver abscesses.

Three research articles—by Holt, Kathryn E; Bialek-Davenet, Suzanne; and Struve, Carsten—focused on genomic sequencing of hvKP. Holt et al. conducted whole-genome sequencing of Klebsiella species, including high-virulence clones (40). Bialek-Davenet et al. developed an openly accessible database, BIGSdb-Kp, to extract medically and epidemiologically relevant information from *Klebsiella pneumoniae* genome sequences (39). Struve et al.’s work emphasized the CC23 clone (50). Li et al.’s retrospective study analyzed the epidemiology, risk factors, and drug resistance of hvKP (51). A review by Carlos Catalan-Najera et al. (52) distinguished between the high-mucosity and high-virulence phenotypes. Zhang et al.’s study examined the geographic distribution, clinical characteristics, and antimicrobial resistance of hvKP in China (45).

In summary, the top 10 most cited articles on hvKP primarily focus on reviews and genomic studies. The high proportion of review articles suggests that hvKP is a research hotspot, with many investigators contributing to the field and achieving significant research depth and breadth. The prominence of genomic studies may be attributed to the critical role of genetics in understanding hvKP’s virulence, drug resistance, and transmissibility. By investigating the genetic makeup of hvKP, scientists can gain deeper insights into its biological characteristics, providing a theoretical foundation for the prevention and treatment of hvKP infections.

### 3.7 Keyword analysis

**Figure 7:**
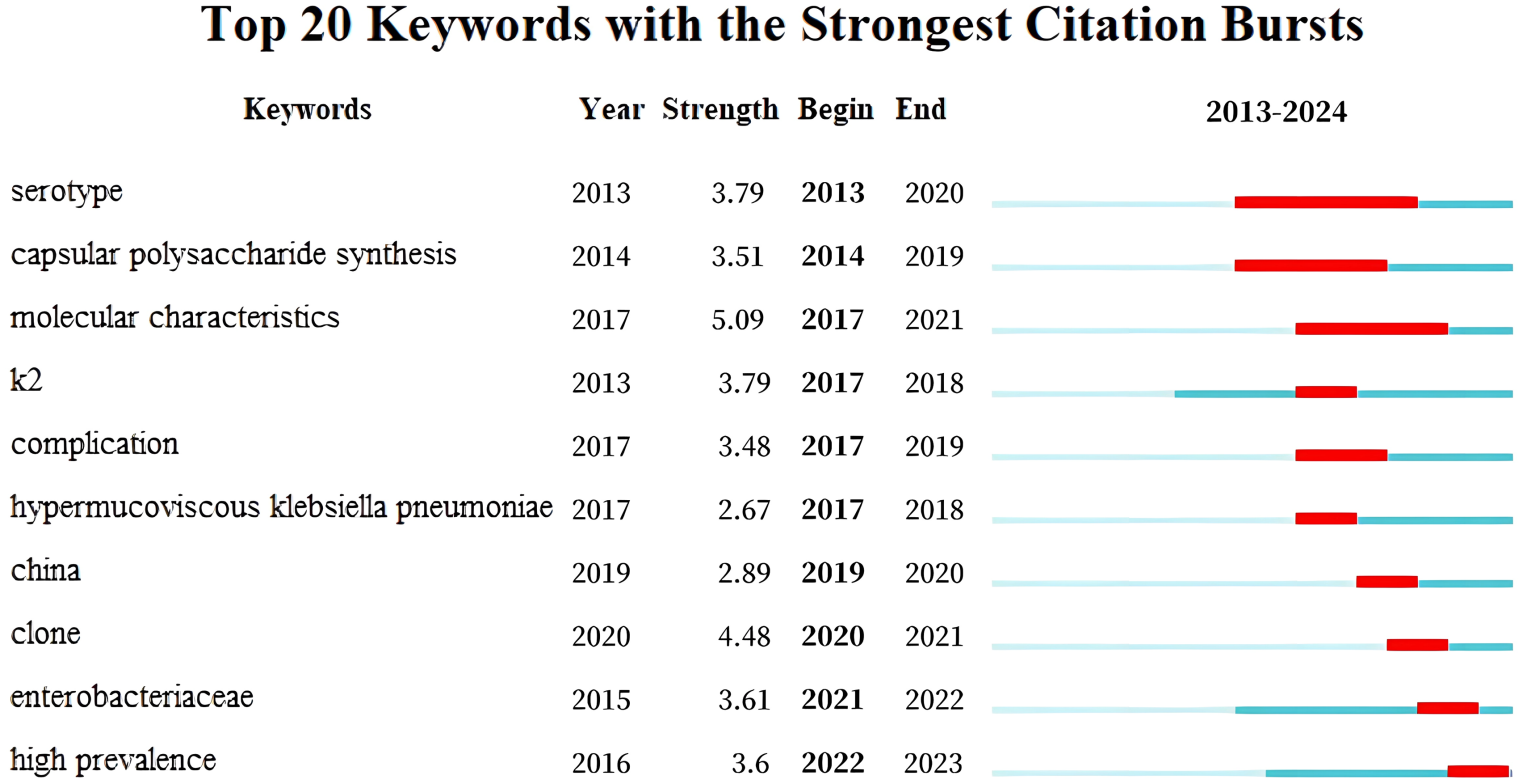
Keyword burst detection.

Supplementary Tables S3a,b list the top 15 keywords by centrality and frequency, respectively. “Pyogenic liver abscess” exhibits a betweenness centrality (BC) of 0.11 > 0.1, highlighting its importance in hvKP research. Figure 7 illustrates keyword burst detection, enabling the division of hvKP research trends into four phases: (2013–2016) Basic Research Phase: focusing on basic biological characteristics (serotype, capsular polysaccharide synthesis, molecular features) and early clinical complications, with initial studies on the K2 gene; (2017–2018) Rise of Hypermucoviscous hvKP: research shifted toward the pathogenesis of hypermucoviscous hvKP, with peak interest in the K2 gene; (2019–2020) Chinese Outbreak and Clone Studies: focus on outbreaks and clones in China, investigating epidemiological characteristics; (2021–2023) Enterobacteriaceae and High Prevalence: research expanded to the entire Enterobacteriaceae family, focusing on the public health impact and control strategies of hvKP.

### 3.8 Key research themes

**Figure 8:**
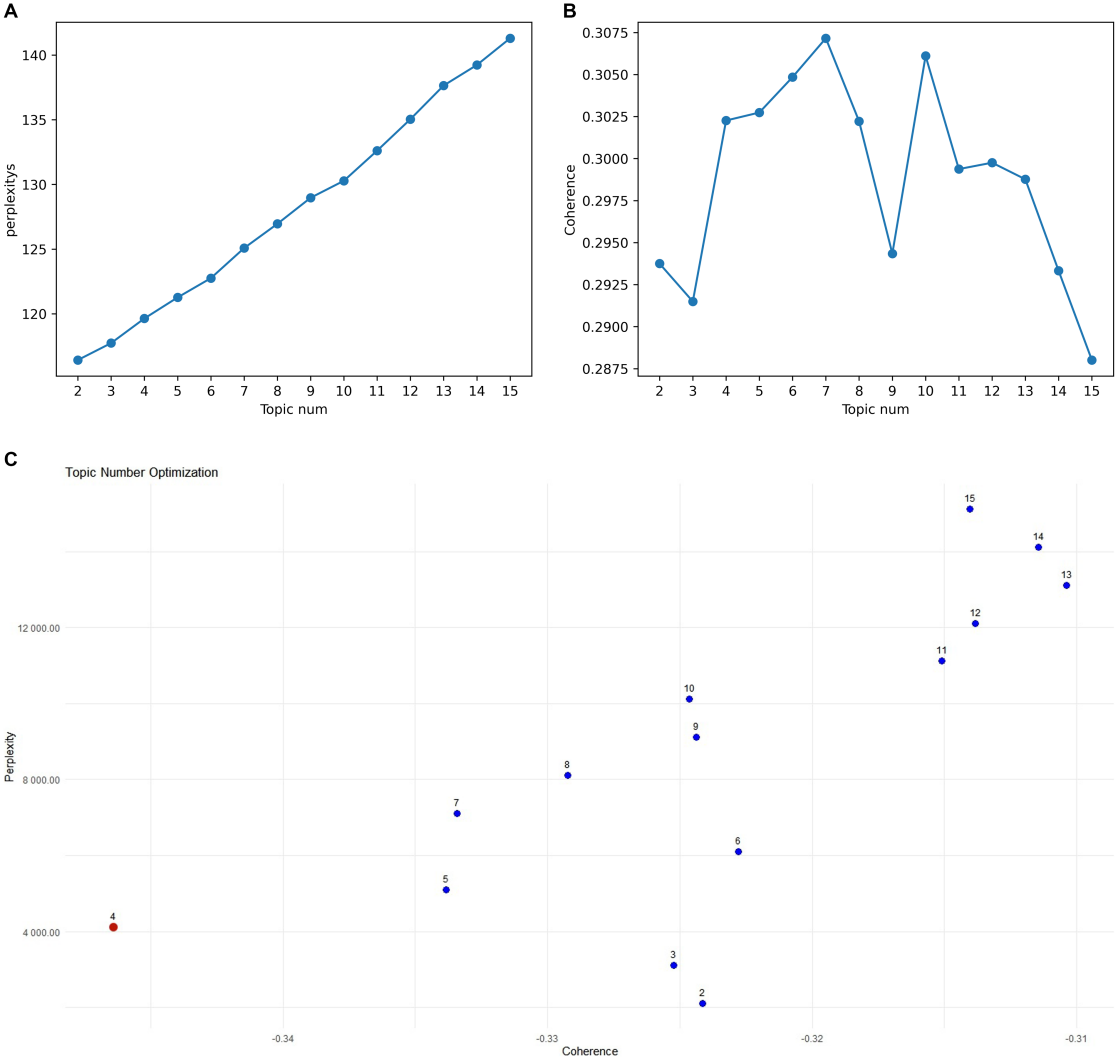
**(A)** Perplexity for topics 2–15. **(B)** Coherence for topics 2–15. **(C)** Topic model optimal parameter selection diagram.

Preprocessing involved removing stop words and punctuation, and stemming to ensure data consistency and cleanliness. The model was trained using the LdaModel class in Gensim, implementing an online LDA algorithm with the following parameter settings: alpha = “symmetric” (symmetric prior) and eta = None (default prior). These parameters control the prior beliefs regarding the document-topic and topic-word distributions. Topic optimization involved training LDA models with varying numbers of topics (2 to 15). Lower perplexity indicates better model fit (Figure 8A), while higher coherence suggests a more coherent topic structure (Figure 8B). The scatter plot in Figure 8C shows that models with 4, 5, and 7 topics balance perplexity and coherence. Further manual analysis indicated that 5 and 7 topics resulted in overly fine-grained and fragmented classifications. A 4-topic model was ultimately selected. Representative keywords for each topic are presented in Supplementary Table S4.

**Figure 9:**
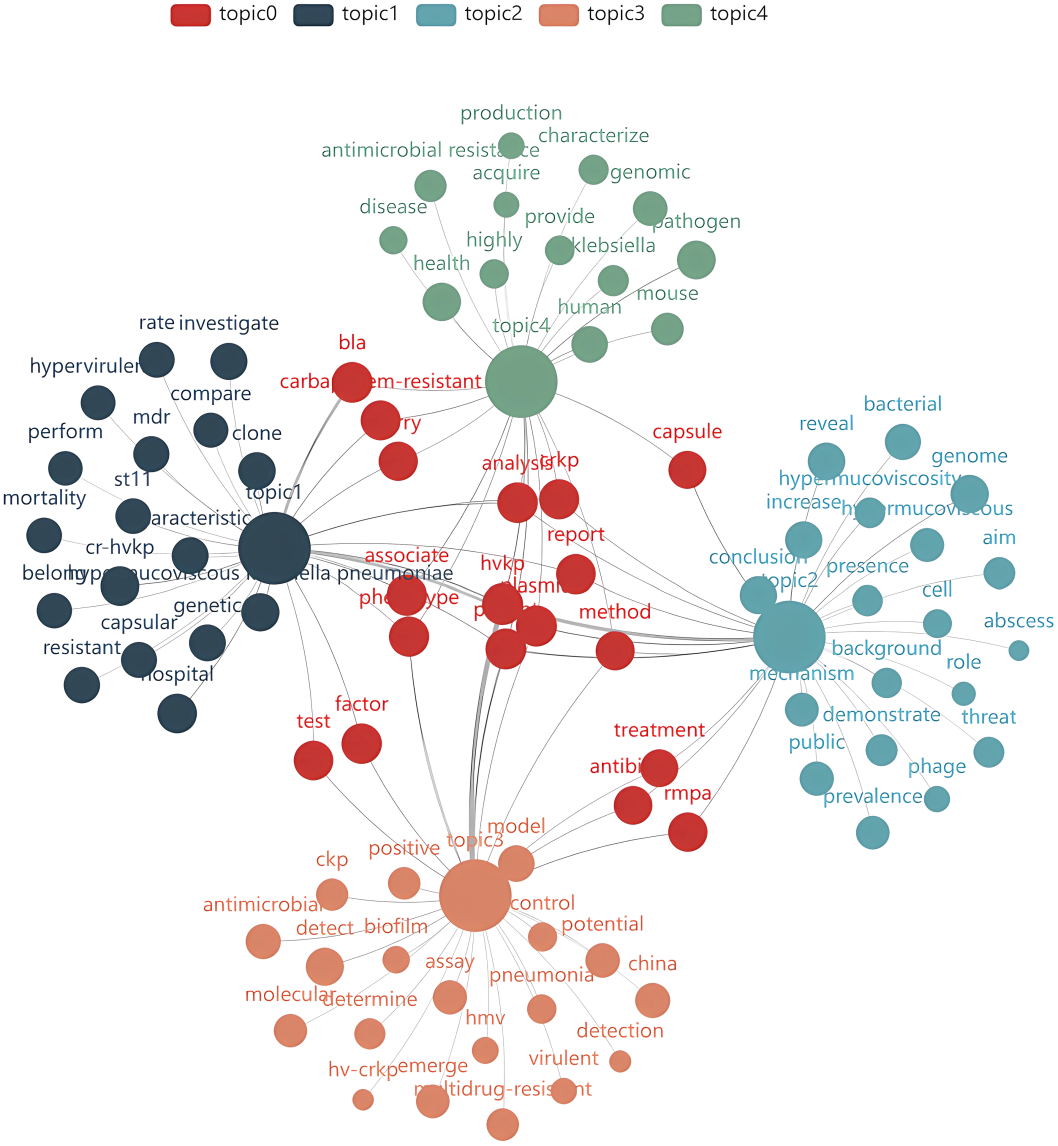
Topic-word relationship diagram.

Figure 9 uses word clouds to visually represent the interrelationships between different research themes, with each cluster marked by a different color. Clusters are interconnected through shared keywords (e.g., red nodes for Topic 0), with node size reflecting keyword frequency and connection thickness representing the strength of word distribution within a specific topic. The analysis reveals significant cross-cluster associations. For example, Cluster 1 (Core Mechanisms and Antimicrobial Resistance) shows a strong association with Cluster 3 (Treatment and Prevention) because the type of resistance genes directly influences antibiotic selection and efficacy. For instance, hvKp strains carrying the NDM-1 gene exhibit high resistance to carbapenem antibiotics (e.g., imipenem). A study on hvKp infections in India found that strains carrying the NDM-1 gene showed resistance rates exceeding 90% against imipenem, leading to a significant increase in treatment failure rates (53). Additionally, Cluster 4 (Epidemiology and Transmission) correlates with Cluster 2 (Clinical Manifestations and Diagnosis) as epidemiological data aids in predicting and preventing infections. For example, patients with a travel history to East Asia should be vigilant about the possibility of hvKP infection (54).

The following sections (3.8.1–3.8.4) delve into these four major topics in depth, exploring their significance and the latest advances in current research.

#### 3.8.1 Phenotype and virulence determinants

*Klebsiella pneumoniae* (hvKP) exhibits hypervirulence primarily due to its virulence factors and phenotypic traits, such as hypermucoviscosity, capsular serotypes (e.g., K1 and K2), virulence genes (e.g., *rmpA*, *rmpA2*, *iucA*, *magA*), high serum resistance, and strong invasiveness (55–57).

##### 3.8.1.1 Hypermucoviscosity

The hypermucoviscous phenotype, a key feature of hvKP, results from increased capsular polysaccharide synthesis (58, 59). This phenotype enhances bacterial virulence by promoting immune evasion, particularly resistance to neutrophil-mediated killing (60). The thickened and sticky capsular polysaccharides form a physical barrier, hindering immune cell recognition and phagocytosis, thereby promoting immune evasion and persistent infection (58, 59). Additionally, hypermucoviscosity may further augment virulence by influencing biofilm formation and bacterial invasiveness. In experimental studies, the hypermucoviscous phenotype is typically detected using the “string test,” where a string length ≥ 0.5 mm indicates positivity (61). This method is simple and widely used in laboratory settings. Furthermore, whole-genome sequencing (WGS) and PCR techniques can identify genes associated with hypermucoviscosity (62). For instance, *rmpA* and *rmpA2* are key regulatory genes for hypermucoviscosity, often located on large plasmids and upregulated to enhance capsular polysaccharide synthesis (58, 59). However, some hvKP strains exhibit hypermucoviscosity in the absence of *rmpA/rmpA2*, suggesting alternative regulatory mechanisms (63). These mechanisms may involve other genes or regulators in the capsular polysaccharide synthesis pathway, warranting further investigation.

##### 3.8.1.2 Capsular serotypes

The capsular serotypes of hvKP, including K1, K2, K5, K20, K54, and K57, are often co-present with virulence genes (e.g., *rmpA*, *rmpA2*, *iucA*), further enhancing their virulence (64, 65). Among these, K1 and K2 are the most extensively studied and prevalent serotypes (66). Khairuddin et al. found that K1 and K2 serotypes accounted for 11.1 and 6.1% of hvKP isolates, respectively (56). These serotypes are frequently associated with severe community-acquired infections, such as liver abscesses, pneumonia, and meningitis (64). In virulence assessments, K1 serotype hvKP demonstrated 100% lethality in the *Galleria mellonella* infection model, while K2 serotype exhibited high serum resistance (67, 68). Whole-genome sequencing has revealed complex relationships between hvKP serotypes and virulence genes. For example, *magA* is a unique virulence gene in the K1 serotype (56), while *rmpA* and *rmpA2* are detected in 99.4 and 98.6% of K1 and K2 serotypes, respectively (69). Additionally, K1 serotype hvKP is often associated with the ST23 sequence type (70, 71). Although K54 and K57 serotypes are less frequent, they have also been reported as virulence markers in some studies (65, 72). In experimental research, PCR is commonly used to detect capsular serotypes and virulence genes; however, variations in detection standards across laboratories may lead to inconsistent results (65).

##### 3.8.1.3 Virulence genes

The virulence genes of hvKP, including *rmpA*, *rmpA2*, *iucA*, *aerobactin*, *iroB*, and *peg-344*, significantly enhance pathogenicity by regulating mucoid phenotypes, iron acquisition systems, and other virulence factors.

- *rmpA* and *rmpA2*: Studies have shown that *rmpA* and *rmpA2* are highly conserved in hvKP and strongly associated with hypervirulence (73–75). For instance, a study in Malaysia reported that all hvKP strains carried *rmpA* and *rmpA2* (56). These genes act as key regulators of capsular synthesis by targeting the promoter of the capsular gene cluster (*cps*), promoting high capsular production and enhancing bacterial adhesion and invasiveness (57, 66, 76). Further research has revealed that the promoter activity of *rmpA* is closely linked to virulence. Strong promoter activity (e.g., P11T and P12T) correlates with high capsular production and invasive virulence, while weak activity (e.g., P9T and P10T) may enhance bacterial colonization. Additionally, mutations (e.g., insertions/deletions) in *rmpA* and *rmpA2* can shift hvKP from a hypermucoid to a hypomucoid phenotype, reducing invasive virulence but enhancing colonization. This adaptive change allows hvKP to persist and spread within hosts, increasing its epidemiological significance (77).
- *iucA* and *aerobactin*: The presence of *iucA* and *aerobactin* in hvKP is strongly associated with hypervirulence, particularly in severe infections such as liver abscesses (78, 79). *iucA* encodes aerobactin synthetase, and aerobactin serves as the primary siderophore for hvKP, facilitating iron acquisition and significantly enhancing bacterial survival and pathogenicity (80). Compared to the unstable *RmpA2*, *aerobactin* exhibits higher stability in carbapenem-resistant hvKP (CR-hvKP) and has been proposed as a reliable marker for hvKP identification (81, 82).
- *iroB*: The *iroB* gene, encoding the salmochelin siderophore system, plays a critical role in iron acquisition, significantly enhancing hvKP survival and pathogenicity (67, 77). Gene knockout studies have demonstrated that *iroB* deletion markedly reduces virulence, confirming its importance in hvKP pathogenicity (83). *iroB* is prevalent in hvKP, a study in Sudan reported a 57.9% detection rate of *iroB* in hvKP clinical isolates (74). Notably, *iroB* often coexists with other virulence genes (e.g., *iucA*, *rmpA2*), which encode siderophores and mucoid phenotype regulators, further augmenting hvKP virulence (84, 85).
- *peg-344*: The presence of peg-344 is strongly correlated with hypervirulence in hvKP. For example, peg-344-positive hvKP strains exhibit enhanced serum resistance, biofilm formation, and invasiveness, as well as increased pathogenicity in animal models (35, 84, 86).

#### 3.8.2 Antibiotic resistance mechanisms

In recent years, the issue of antibiotic resistance in hypervirulent *Klebsiella pneumoniae* (hvKP) has become increasingly severe, particularly with the emergence of multidrug-resistant (MDR) and carbapenem-resistant (CRKP) strains, posing significant challenges to clinical treatment (55, 87). The development of novel antibiotics and vaccines targeting hvKP, as well as the use of gene-editing technologies such as CRISPR-Cas9 to knockout resistance genes, are likely to be future research hotspots (88, 89). Below, we systematically elucidate the resistance mechanisms of hvKP from two perspectives: antibiotic classification and molecular mechanisms.

Resistance mechanisms in hvKP involve outer membrane porin mutations, plasmid-mediated gene transfer, efflux pumps, and biofilm formation. Mutations in outer membrane porins (such as ompK35 and ompK36) reduce the ability of antibiotics to enter bacterial cells, thereby enhancing resistance. Zhao et al. (90) found that the L359R mutation in ompK36 is associated with hvKP’s resistance to ceftazidime/avibactam. Additionally, hvKP carries multiple resistance genes (such as blaCTX-M, blaTEM, blaSHV, etc.) via plasmids and spreads resistance among strains through conjugation (90–92). The efflux pump system (e.g., acrAB and tolC) actively expels antibiotics from the cell, reducing their intracellular concentration and thereby enhancing hvKP’s resistance to multiple antibiotics (93). Alharbi et al. (94) noted that hvKP protects itself by forming biofilms, increasing its tolerance to antibiotics.

The antibiotic classification-based resistance mechanisms involve carbapenems, colistin, fosfomycin, and multidrug resistance mechanisms. Carbapenem resistance is mainly mediated by the spread of carbapenemase genes (such as blaKPC and blaNDM), which are transferred via plasmids or chromosomes, conferring hvKP resistance to carbapenems (95–97). Liu et al. (95) demonstrated that the blaKPC gene spreads via IncFII plasmids in ST11-type hvKP, while Tang et al. (97) showed that the blaNDM-1 gene can be horizontally transferred through outer membrane vesicles (OMVs). For instance, Liu et al. (95) found that the blaKPC gene spreads through IncFII plasmids in ST11-type hvKP, while Tang et al. (97) confirmed that the blaNDM-1 gene can be horizontally transferred in hvKP via outer membrane vesicles (OMVs). Colistin resistance is primarily associated with mgrB gene mutations and overexpression of the phoPQ system, which modify the lipopolysaccharide (LPS) structure of the bacterial outer membrane, reducing colistin binding capacity and thereby inducing resistance (87). Intrinsic mechanisms of fosfomycin resistance include the UhpTE350Q mutation and the presence of fosA6/5 genes, which affect fosfomycin uptake or metabolism, leading to hvKP resistance to fosfomycin. Furthermore, hvKP achieves multidrug resistance and high virulence by carrying multiple resistance genes (such as blaKPC, blaNDM, blaOXA, etc.) and virulence genes (such as *rmpA*, *iucA*, etc.) (98, 99). For example, Zhang et al. (100) reported a hybrid plasmid in ST11-KL64 type hvKP carrying blaKPC-2 and *rmpA2* genes, indicating the co-evolution of resistance and virulence genes.

#### 3.8.3 Genomic detection approaches

Traditional culture and biochemical identification methods, while reliable, are limited in terms of timeliness and sensitivity. With the rapid development of molecular biology techniques, genome-based detection methods have gradually become mainstream, leading to significant progress in the genomic research of hvKp. This has unveiled its virulence mechanisms, evolutionary pathways, and drug resistance characteristics (101, 102).

##### 3.8.3.1 Research methods

- *Whole genome sequencing*: WGS is a core technology for the classification of hvKP. By sequencing the entire genome of hvKP strains, researchers can identify virulence-associated genes and mutation sites, providing comprehensive genomic information. For example, by comparing the genomes of different hvKP strains, high-virulence-associated gene clusters such as *rmpA* and *rmpA2* can be identified. This method offers crucial insights for the classification and evolutionary studies of hvKP (103, 104).
- *Single nucleotide polymorphism analysis*: SNP analysis is another commonly used genomic detection method. By comparing SNP sites in hvKP strains, phylogenetic trees can be constructed, enabling strain classification and evolutionary analysis. This approach is particularly valuable for tracing the transmission routes and evolutionary relationships of hvKP (105, 106).
- *Machine learning algorithms*: In recent years, machine learning algorithms have seen increasing application in genomic data analysis. By training deep learning models, researchers can automatically identify hvKP features and perform classification from high-throughput genomic data. For instance, models based on convolutional neural networks (CNN) and long short-term memory networks (LSTM) have demonstrated high accuracy in hvKP classification (107, 108).

##### 3.8.3.2 Application directions

- *Virulence gene detection*: The detection of virulence genes is a critical basis for hvKP classification. Using PCR or high-throughput sequencing, researchers can identify virulence-associated genes such as *rmpA*, *aerobactin*, and iroN. The presence or absence of these genes directly reflects the virulence level of the strains (103, 104).
- *Genomic evolution and diversity*: Through whole-genome sequencing and comparative genomic analysis, hvKp has been classified into multiple clonal lineages, such as ST23, ST65, and ST86, with ST23-K1 and ST86-K2 being the predominant hvKp clonal lineages (101, 102).
- *Drug resistance mechanism research*: hvKp has increasingly exhibited resistance to multiple antibiotics, particularly carbapenems, which is closely associated with the presence of resistance genes such as blaKPC and blaNDM (101, 109).

#### 3.8.4 Clinical epidemiology

The clinical epidemiology of hypervirulent *Klebsiella pneumoniae* (hvKP) is characterized by its high-risk populations, infection types, low resistance but high virulence, and transmission patterns. hvKP primarily affects individuals with diabetes, long-term hospitalized patients, and immunocompromised populations, leading to severe infections such as pyogenic liver abscess (PLA) and ventilator-associated pneumonia (VAP) (110). Although hvKP exhibits lower antibiotic resistance compared to carbapenem-resistant *Klebsiella pneumoniae* (CRKP), its virulence is significantly higher, particularly in strains carrying virulence genes such as iucA and rmpA and those of the ST23 lineage, which are associated with a hypermucoid phenotype and severe infections (111).

The transmission of hvKP is closely linked to healthcare settings, especially in long-term care facilities and intensive care units, where it poses a significant threat to vulnerable populations Additionally, the increasing incidence of community-acquired hvKP infections highlights its ability to spread beyond hospital environments, further complicating its control and management (112). Studies have shown that diabetic patients and those with gallstones are particularly susceptible to hvKP-related PLA, while elderly patients and those with pressure ulcers in long-term care facilities are at higher risk of hvKP infections (110, 113).

Research methodologies such as retrospective analyses, genomic studies, and phenotypic experiments have been instrumental in understanding hvKP’s epidemiology. For instance, Guo et al. used genomic analysis to differentiate between PLA- and VAP-associated strains (110), while Alfaifi et al. conducted retrospective studies to examine resistance patterns in long-term care settings. However, limitations such as small sample sizes and regional focus in some studies may affect the generalizability of findings (113). Further research is needed to elucidate hvKP’s transmission mechanisms, its interactions with host immunity, and the development of novel therapeutic strategies, particularly in the context of its increasing prevalence in Asia and its potential crossover with CRKP (110, 112, 113).

## 4 Study limitations

This study has the following limitations.

1. *Database selection*: This study exclusively used the Web of Science (WoS) database as the data source and did not include other important databases such as PubMed/MEDLINE, Cochrane, or Embase/SCOPUS. This may have resulted in the omission of some relevant literature, particularly clinical and regional studies. Future research should consider incorporating more databases to ensure comprehensive coverage of the literature.
2. *Limitations of topic modeling*: Although topic modeling can reveal the latent structure of research themes, its results depend on text preprocessing and model parameter selection. The Latent Dirichlet Allocation (LDA) model used in this study, while effective in identifying topics, may have been influenced by text cleaning, stop-word removal, and stemming processes. Additionally, the choice of the number of topics is somewhat subjective and may affect the final topic classification.

Despite these limitations, this study provides a systematic analytical framework for understanding the knowledge structure and research trends in the field of hvKP through bibliometric and topic modeling methods, offering valuable insights for future research.

## 5 Conclusion

This study comprehensively reviewed the progress in the field of hypervirulent *Klebsiella pneumoniae* (hvKP) research through bibliometric analysis and topic modeling methods. The results indicate that hvKP research has shown significant growth over the past decade, particularly in Asia, with China emerging as the primary contributor in this field. Research hotspots primarily focus on hvKP’s phenotypic and virulence determinants, antibiotic resistance mechanisms, genomic detection methods, and clinical epidemiology. Through bibliometric analysis, we identified the formation of collaborative networks among core authors, institutions, and countries, with particularly strong collaborations between China, the United States, and France. Additionally, topic modeling revealed major research directions, including the identification of virulence genes, the elucidation of resistance mechanisms, and the application of genomics in hvKP classification and evolutionary studies.

Despite significant advancements, hvKP research still faces numerous challenges. Firstly, the precise definition and diagnostic criteria for hvKP remain inconsistent, making it difficult to directly compare results across different studies. Secondly, the mechanisms of hvKP virulence and the evolution of resistance are complex, requiring further in-depth research to uncover their molecular basis. Moreover, the global transmission trends and epidemiological characteristics of hvKP still need to be validated through large-scale, multicenter studies. Future research should focus on the genomic evolution of hvKP, the co-evolution of resistance and virulence, and the development of novel therapeutic strategies, particularly for multidrug-resistant hvKP strains.

## Data Availability

The original contributions presented in the study are included in the article/Supplementary material, further inquiries can be directed to the corresponding authors.
