# Supplementary material for: A systematic review of hypervirulent Klebsiella pneumoniae research: bibliometric and topic modeling perspectives
Source: Front Med (Lausanne). 2025 Apr 11;12:1545678. doi: 10.3389/fmed.2025.1545678 (PMC12021593; doi:10.3389/fmed.2025.1545678)
Supplement: Supplementary file 1 [file Table_1.docx]

**Supplementary Material**

**Table S1:** Top 10 Journals in hvKP Research

*（Ranked by Number of Publications (NP), Along with Their H-Index, Impact Factor (IF), and Journal Citation Reports (JCR) Category）*

| Journal | NP | H-index | IF | JCR |  |
| --- | --- | --- | --- | --- | --- |
| Frontiers in Microbiology | 57 | 16 | 5.2 | Q2 |  |
| Infection and Drug Resistance | 46 | 10 | 3.9 | Q2 |  |
| Frontiers in Cellular and Infection Microbiology | 38 | 14 | 5.7 | Q1 |  |
| Microbiology Spectrum | 24 | 5 | 3.7 | Q2 |  |
| Antimicrobial Agents and Chemotherapy | 19 | 13 | 4.9 | Q2 |  |
| Microbial Drug Resistance | 19 | 9 | 2.6 | Q3 |  |
| Journal of Global Antimicrobial Resistance | 19 | 8 | 4.2 | Q2 |  |
| Microorganisms | 19 | 7 | 4.5 | Q2 |  |
| Journal of Antimicrobial Chemotherapy | 15 | 10 | 5.2 | Q2 |  |
| Scientific Reports | 14 | 8 | 4.6 | Q2 |  |

**Table S2:** Top 10 most cited publications ranked by Local Citation Score (LCS)

| Paper Title | Author | Year | LCS |
| --- | --- | --- | --- |
| Hypervirulent (hypermucoviscous) *Klebsiella pneumoniae*: a new and dangerous breed | Russo, Thomas A | 2013 | 350 |
| Hypervirulent *Klebsiella pneumoniae* | Russo, Thomas A | 2019 | 205 |
| *Klebsiella pneumoniae*: Going on the Offense with a Strong Defense | Paczosa, Michelle K | 2016 | 185 |
| *Klebsiella pneumoniae* liver abscess: a new invasive syndrome | Siu, L. Kristopher | 2012 | 164 |
| Genomic analysis of diversity, population structure, virulence, and antimicrobial resistance in *Klebsiella pneumoniae*, an urgent threat to public health | Holt, Kathryn E | 2015 | 146 |

**Table S3.** High-frequency keyword analysis in hvKp research

1. Ranking by betweenness centrality (BC)

*（BC measures keyword connectivity in co-occurrence networks）*

| Keyword | Centrality | Count |
| --- | --- | --- |
| escherichia coli | 0.16 | 120 |
| pyogenic liver absce | 0.11 | 60 |
| gene | 0.07 | 116 |
| epidemiology | 0.07 | 71 |
| identification | 0.07 | 54 |
| beta lactamase | 0.07 | 44 |
| Hypermucoviscous *k. pneumoniae* | 0.07 | 13 |
| strain | 0.06 | 114 |
| serotype k1 | 0.06 | 67 |
| bacteremia | 0.06 | 51 |
| association | 0.06 | 37 |
| *klebsiella pneumoniae* | 0.05 | 324 |
| liver absce | 0.05 | 169 |
| antimicrobial resistance | 0.05 | 89 |
| hypervirulent | 0.05 | 84 |

*(b) Ranking by publication frequency*

| Keyword | Count | Centrality |
| --- | --- | --- |
| *klebsiella pneumoniae* | 324 | 0.05 |
| liver abscess | 169 | 0.05 |
| virulence | 160 | 0.02 |
| infection | 153 | 0.03 |
| escherichia coli | 120 | 0.16 |
| gene | 116 | 0.07 |
| strain | 114 | 0.06 |
| emergence | 112 | 0.04 |
| antimicrobial resistance | 89 | 0.05 |
| hypervirulent | 84 | 0.05 |
| resistance | 84 | 0.04 |
| epidemiology | 71 | 0.07 |
| k1 | 68 | 0.05 |
| serotype k1 | 67 | 0.06 |
| enterobacteriaceae | 64 | 0.03 |

**Table S4. Topic-keyword distributions from LDA modeling**

| Topic interpretation | Representative keywords |
| --- | --- |
| 1：Phenotype & Virulence Determinants | phenotype;hypervirulence;capsular;virulence plasmid |
| 2：Antibiotic Resistance Mechanisms | plasmid;carbapenem-resistant;antibiotic resistance;multidrug-resistant;antibiotic;resistance |
| 3：Genomic Detection Approaches | genome;sequencing;genetic;molecular;assay;detection |
| 4：Clinical Epidemiology | patient;hospital;surveillance;transmission;outbreak;prevalence |

*Note: Topics were generated using LDA (Latent Dirichlet Allocation) modeling. Keywords were filtered through the following steps:*

*1. Low-frequency filtering: Excluded terms below the 80th percentile of cumulative token frequency (retaining the top 20% of total token occurrences).*

*2. High-frequency filtering: Removed terms appearing in >40% of documents to discard oversaturated terms.*

*3. Manual screening: Excluded irrelevant keywords, selected dominant keywords, and assigned topic names based on keyword types.*
